# Analysis of Clinicopathological Factors Influencing Survival in Patients with Renal Cell Carcinoma and Venous Tumor Thrombus

**DOI:** 10.3390/jcm10173852

**Published:** 2021-08-27

**Authors:** Łukasz Zapała, Sumit Sharma, Michał Kunc, Piotr Zapała, Jakub Kłącz, Piotr Korczyński, Michał Lipowski, Michał Późniak, Tomasz Suchojad, Tomasz Drewa, Marcin Matuszewski, Piotr Radziszewski

**Affiliations:** 1 Clinic of General, Oncological and Functional Urology, Medical University of Warsaw, 02-005 Warsaw, Poland; sumit.sharma29.06.91@gmail.com (S.S.); zapala.piotrek@gmail.com (P.Z.); pradziszewski@wum.edu.pl (P.R.); 2 Department of Pathomorphology, Medical University of Gdańsk, 80-214 Gdańsk, Poland; 3 Department of Urology, Faculty of Medicine, Medical University of Gdańsk, 80-402 Gdańsk, Poland; k.kuba@gumed.edu.pl (J.K.); matmar@gumed.edu.pl (M.M.); 4 Department of Urology, Regional Specialist Hospital, 26-060 Czerwona Góra, Poland; pwkorczynski@gmail.com (P.K.); tritomek@gmail.com (T.S.); 5 Department of Urology, St. Lukas Specialist Hospital, 26-200 Końskie, Poland; michal1978eu@gmail.com; 6 Clinic of Urology, Dr Jurasz University Hospital, 85-094 Bydgoszcz, Poland; michalpozniak90@gmail.com (M.P.); t.drewa@wp.pl (T.D.)

**Keywords:** renal cell cancer, tumor thrombus, radical nephrectomy, prognostic factors

## Abstract

This study aimed to define patients with renal cell cancer and coexisting tumor thrombus in order to address concerns regarding survival and prognostic factors after radical surgery. Several prognostic factors for overall survival (OS) were assessed in patients treated surgically at five institutions from 2012 to 2018. Univariate and multivariate analyses were used to determine the independent risk factors of OS. A total of 142 patients were eligible for further analysis (mean age of 64.75 years, 56% males). Most patients presented with clear cell carcinoma (95%). The Mayo stage was predominantly 0–1 (88%). Distant visceral metastases at the time of diagnosis were present in 36 patients (25%), whereas nodal metastases were present in 24 patients (16.9%). During the follow-up period (mean of 32.5 months), the 3-year OS rate reached 68.2%. The majority of patients received no adjuvant treatment (*n* = 107). In a multivariable model predicting OS, regional lymph node status (*p* < 0.001), distant metastases (*p* = 0.009), tumor grade (*p* = 0.002), duration of hospitalization (*p* = 0.016), and Clavien–Dindo grade (*p* = 0.047) were identified as independent prognostic factors. A subgroup of patients with specific clinicopathological factors may benefit most from the radical surgery, including patients without regional lymph node or distant metastases and with low tumor grades, whereas short hospitalization and low Clavien–Dindo grades represent additional independent prognostic factors.

## 1. Introduction

The currently available data on renal cell carcinoma (RCC) with tumor thrombus are inconclusive in terms of the optimal management of such cases [1]. The treatment of patients with no distant metastases comprises a complicated surgical procedure of radical nephrectomy combined with cavotomy and thrombectomy, with early complications present in 58% of cases, out of which 30% are severe, including death [2].

RCC presents with tumor thrombus in up to 10% of cases [3]. The authors emphasize the existence of two major subtypes: with invasion restricted to the renal vein, or with distal propagation to the inferior vena cava and right atrium [1]. The cases with tumor thrombus confined to the renal vein are frequently treated in non-tertiary referral centers and may encompass up to 78% of T3 disease [4]. In many clinical scenarios, the management of such cases includes a simple thrombectomy without caval reconstruction [5], and surgery does not require cardiac arrest with extracorporeal circulation [1]. Thus, patient selection seems to be of crucial importance and should be based on a variety of prognostic factors.

The majority of papers claim that the presence of tumor thrombus is associated with less favorable survival rates when compared to other RCC cases [3,4]. When analyzing long-term overall survival (OS) in a group of patients with tumor thrombus, one should focus not only on clinical but also on pathological features, and, finally, on the specific characteristics of tumor thrombus [1]. The development of targeted treatment has improved the prognosis of patients with metastatic RCC [6]. As a consequence, some authors emphasize that risk stratification may facilitate the selection of patients who will benefit from adjuvant systemic treatment in non-metastatic disease [1,7]. Finally, based on the current data, the 5-year OS rate varies from 34% to 71% [1,5,8,9].

Therefore, future studies are needed with a special interest in the prognostic risk factors in this heterogeneous group of patients. In the current multi-institutional retrospective series, we have analyzed the potential prognostic value of various clinicopathological factors in a cohort of patients with RCC and coexisting tumor thrombus treated with radical surgery.

## 2. Materials and Methods

### 2.1. Study Group

Medical records of 172 patients with pathologically confirmed RCC with venous thrombus who had undergone nephrectomy with or without cavotomy and thrombectomy in the years 2012–2018 in 5 urological centers (three tertiary referral centers and two non-tertiary referral centers) were retrieved from local medical databases. Nephrectomy with or without cavotomy and thrombectomy was performed using standard methods for open radical nephrectomy via lumbotomy or laparotomy. Lymph node dissection was performed in all cN1 cases. The following data were collected: (a) demographic: age, gender; (b) clinical: length of hospitalization, American Society of Anesthesiologists scale (ASA), blood transfusions, staging based on CT or MRI scans of chest, abdomen, and pelvis according to 2017 TNM classification system [10], staging according to the classification of tumor thrombus level according to the Mayo staging system, as described before [11,12], adjuvant systemic treatment; and (c) pathological: histological diagnosis including grade (according to Fuhrman and/or WHO/ISUP when adequate), presence of necrosis within the tumor, as well as the dates of diagnosis and death, and the last follow-up. ASA was assessed according to Little P. [13]. OS was defined as the time from the nephrectomy to death from any cause. All the patients enrolled had no additional treatments before radical nephrectomy. Additionally, telemedicine visits were recorded when considering follow-up details. Thirty patients (*n* = 30) were excluded from the study due to incomplete clinicopathological and survival data (lost to follow-up after surgery), leaving 142 patients in the final group. The major reason for the loss to follow-up was patients finishing their main treatment in the cancer centers engaged in the study and moving to local/regional urological centers or outpatient clinics. Local contact details were usually incomplete or not valid in the cases lost to follow-up. These cases were random and in the authors’ opinion do not represent a selection bias.

### 2.2. Statistical Analysis

Univariate and multivariate Cox proportional hazards regression (for long-term OS analysis) and logistic regression (to assess factors associated with short-term mortality) were performed. The backward selection was employed to create a multivariable model predicting death and to eliminate non-significant variables at *p* < 0.05. Differences in OS between groups were assessed using the log-rank test and visualized with Kaplan–Meier curves. Statistical analysis was performed with Statistica 13.3 software (TIBCO Software Inc., Palo Alto, CA, USA) and R statistical environment [14]. Kaplan–Meier curves were plotted using the “survminer” and “ggsci” packages [15,16].

## 3. Results

### 3.1. Baseline Characteristics

The baseline characteristics of the study group are shown in Table 1. The mean age of patients was 64.75 years (median = 66; SD = 10.92; range = 31–86 years) and males represented 56% of cases. The mean maximal renal tumor size was 76.05 mm (median = 72; SD = 31.98; range = 10–200 mm). The majority of patients presented with clear cell carcinoma (95%). Predominantly, the Mayo stage in the studied group was 0–1 (88%). Usually, the procedure was conducted via laparotomy, i.e., in 84/142 (59%) cases. The vast majority of cases developed only minor complications: 113/142 (79%) of grade I and 5/142 (3%) of grade II. No cases of dislodgement of the tumor thrombus were noted intraoperatively and no intraoperative deaths were seen. The mean length of hospitalization was 10 days (median = 8; SD = 6.71; range = 3–66 days). In the studied cohort, most patients presented with ASA I–II (*n* = 127, 78.16%, Table 1). Most patients (*n* = 92, 64.8%) were free of both distant and regional nodal metastases, 26 (18.3%) patients had only distant metastases, 14 (9.9%) patients had only nodal metastases, and 10 (7%) patients presented with both nodal and distant metastases. During the follow-up period, the majority of patients received no adjuvant treatment (*n* = 107, 75%), while 1 received cytokine therapy, 29 (20%) received tyrosine kinase inhibitors, and 5 (3%) received monoclonal antibodies.

### 3.2. Survival Analysis

**Figure 1:**
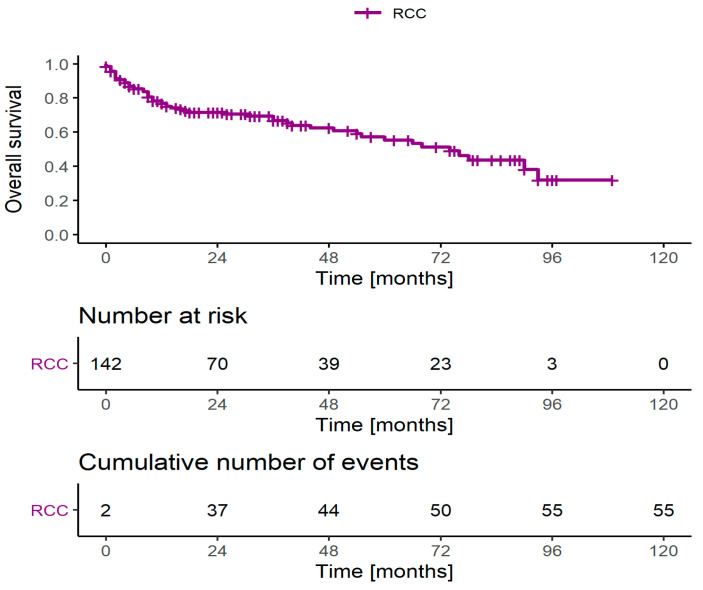
Overall survival for the patients with RCC and tumor thrombus. Abbreviations: RCC: renal cell carcinoma.

The mean follow-up time (calculated for all censored and completed observations) was 32.5 months (median = 22.5 months), while the 3-year OS rate reached 68.2% (Figure 1).

#### 3.2.1. Demographical Data

No difference was found regarding OS between males and females (*p* = 0.5, log-rank), or between groups stratified by age (cut-off 65 years; *p* = 0.2, log-rank).

#### 3.2.2. Clinical Data

**Figure 2:**
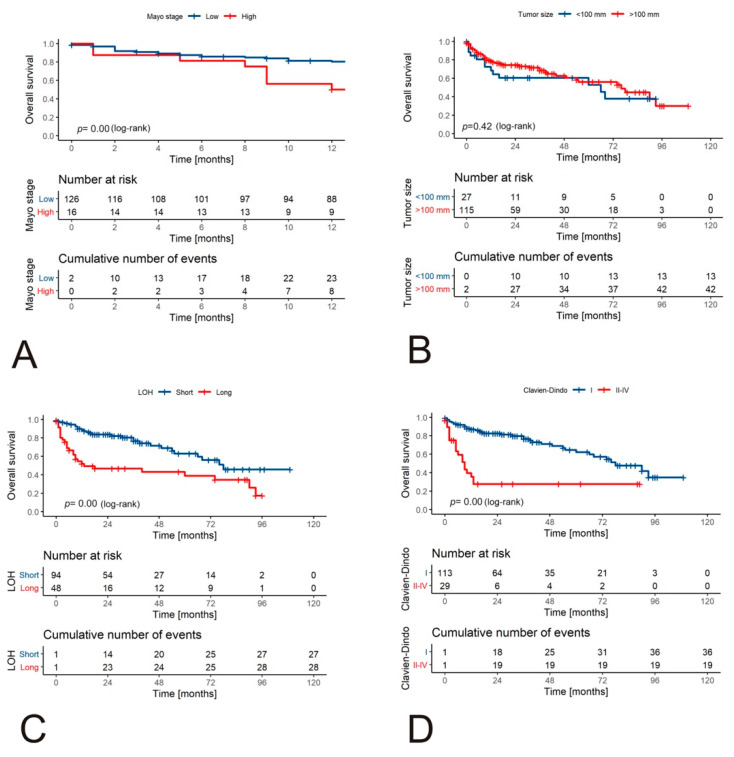

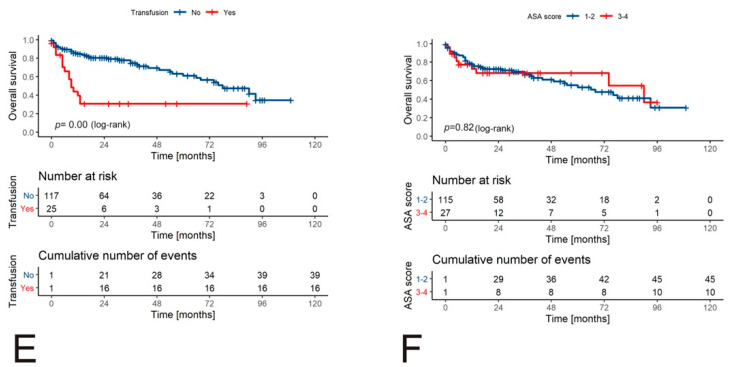
Survival of patients with RCC and tumor venous thrombus according to clinical variables. The Kaplan–Meier curves for one-year OS stratified by Mayo stage (**A**) and long-term OS (**B**–**F**). OS stratified by Mayo stage (**A**), tumor size (**B**), LOH (**C**), Clavien–Dindo classification (**D**), blood transfusion (**E**), and ASA (**F**). Abbreviations: RCC: renal cell carcinoma; OS: overall survival; LOH: length of hospitalization (short defined as ≤9 days); ASA: American Society of Anesthesiologists scale.

A short LOH (defined as ≤9 days) was associated with a more favorable OS rate when compared to a long one (*p* = 0.0000) (Figure 2C). Similarly, patients with a Clavien–Dindo complication rate >I showed a worse OS rate (*p* < 0.0001, log-rank). Taking into consideration perioperative blood transfusions, no intervention was associated with a better OS rate (*p* < 0.0001, log-rank) (Figure 2D). Mayo stage (*p* = 0.16, log-rank), tumor size >10 cm (*p* = 0.42, log-rank) (Figure 2B), and ASA (*p* = 0.82, log-rank) (Figure 2F) were not significantly associated with long-term survival rate. Importantly, the high Mayo stage was associated with a high risk of death during the first 12 months after surgery (log-rank *p* = 0.0075, Figure 2A). This phenomenon may be associated with the fact that the high Mayo stage was associated with longer hospitalization (*p* = 0.003, Mann–Whitney U test), blood transfusions (*p* = 0.0003, Chi-square), and high-grade Clavien–Dindo classification (*p* = 0.0002, Chi-square).

Finally, no differences in OS rate were observed regarding centers, i.e., between tertiary referral hospitals (academic centers) and lower referral hospitals (non-academic centers) (*p* = 0.57, log-rank). Additional calculations revealed no statistically significant differences regarding the distribution of Mayo stages in various centers.

#### 3.2.3. Pathological Data

**Figure 3:**
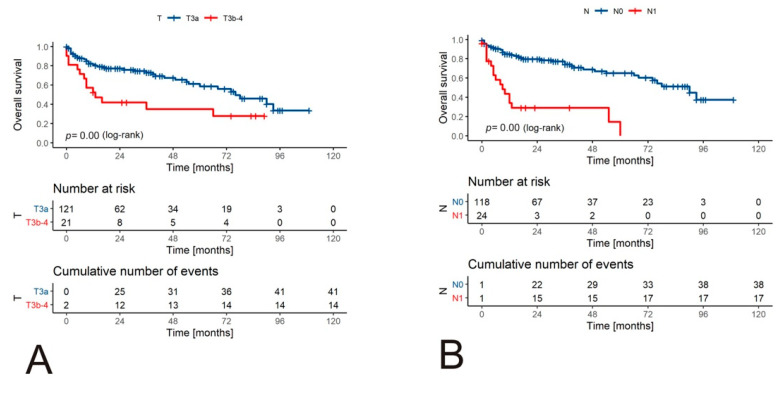

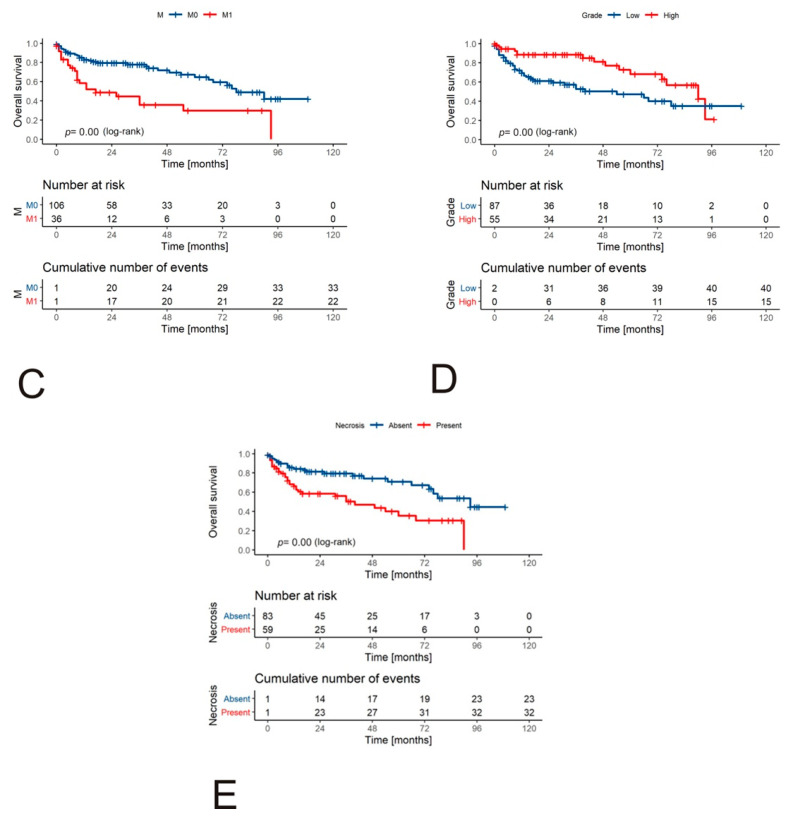
Survival of patients with RCC and tumor venous thrombus according to pathological variables. The Kaplan–Meier curves for OS are stratified by T descriptor (TNM 8th edition) (**A**), regional lymph nodes status; (**B**), the presence of distant metastases; (**C**), tumor grade; (**D**), and presence of necrosis within the tumor (**E**).

Patients with a pathological stage >T3a had a worse OS rate when compared to T3a cases (*p* = 0.004, log-rank) (Figure 3A–E). Similarly, the presence of metastases in regional lymph nodes (*p* < 0.001, log-rank), distant metastases (*p* < 0.001, log-rank), high-grade histology (defined as ≥G3, *p* = 0.0063, log-rank), and the presence of tumor necrosis (*p* < 0.001, log-rank) were associated with inferior OS.

#### 3.2.4. Long-Term and Short-Term Multivariate Survival Analysis

Based on the available data, the univariate and multivariate Cox regression analyses of factors predicting OS were performed (Table 2). The multivariable mortality risk model incorporated regional lymph node status (*p* < 0.001), distant metastases (*p* = 0.009), tumor grade (*p* = 0.002), duration of hospitalization (*p* = 0.016), and Clavien–Dindo grade (*p* = 0.047). Furthermore, in the univariate analysis the following groups of patients were of poorer prognosis: those who received blood transfusions (*p* < 0.001); those with >T3a tumors (*p* = 0.005); those who underwent non-radical procedures, i.e., ≥R1 in the pathology report (*p* = 0.027); and those with tumor necrosis in the specimen (*p* < 0.001). However, these factors did not retain their significance in the multivariate analysis (Table 2).

As a substantial portion of the patients died during the first year of the follow-up, we also aimed to investigate the factors influencing short-term mortality. The 30- or 90-day and 12-month mortality rates were 3.5% (*n* = 5/142), 8.45% (*n* = 12/142), and 22.53% (*n* = 32/142), respectively. Due to the very low number of cases with 30-day mortality, we performed additional analyses only for 90-day and 12-month mortality rates. In the multivariate logistic regression analysis, the risk of 90-day mortality was significantly associated with long hospitalization (OR = 7.0, 95% CI = 1.80–27.26, *p* = 0.005). On the other hand, 12-month mortality was associated with Clavien–Dindo grade (OR = 4.82, 95% CI = 1.85–12.59, *p* = 0.001), tumor necrosis (OR = 0.403, 95% CI = 0.20–0.80, *p* = 0.01), and grade (OR = 0.121, 95% CI = 0.05–0.29, *p* = 0.000). Mayo stage was associated with one-year mortality, but only in the univariate analysis (OR = 4.25, 95% CI = 0.14–12.47, *p* = 0.008).

## 4. Discussion

In light of the existing evidence, the most potent curative management of advanced renal cancer appears to be a surgical resection [11]. However, not all patients are suitable for radical surgery and, even if implemented, the prospects for long-term survival remain dismal [17]. Thus, the prognostication of outcomes of RCC with tumor thrombus represents a major concern, especially in more advanced or complicated cases [18]. Some prognostic factors applicable for renal cancer, i.e., tumor size, grading, histological subtype, presence of sarcomatoid features, invasion of perirenal tissues, and nodal or distant metastasis, have been validated in the case of tumor thrombus [19]. The available data suggest that the successful removal of the tumor thrombus improves survival [20]. In the present study, all the tumor thrombi were successfully removed during the procedure and no intraoperative deaths were noted. Other authors have observed similar results; however, the risk of surgery-related death was 2.8% [21]. The frequency of possibly fatal thromboembolism is estimated to be 5.7% in this population [22].

In the current cohort of patients, the 3-year OS rate reached 68.2%. Based on 292 cases, Hirono et al. reported a 47.6% 5-year OS rate [21], while in the cohort reported by Tang et al. it was 53.6% (*n* = 169) [23]. Reports on cases with tumor thrombus restricted to the renal vein have claimed an even better 5-year OS rate [1]. Some of the differences in the reported survival rates may be due to differences in follow-up, center experience in the treatment of RCC with tumor thrombus, and the level of hospitals recruiting patients [4,18,21]. Ficarra et al. found that renal vein or subdiaphragmatic inferior vena cava involvement did not influence patients’ survival when compared to individuals with clinical T2N0M0 disease if there was no perirenal fat invasion, or lymph node or distant metastases [24].

Unfortunately, even patients at the M0 stage at the time of surgery may finally deteriorate due to the development of distant metastases. This phenomenon may be explained by the process of tumor cell seeding via the venous system, the existence of residual cancer cells in the venous wall, or the presence of clinically occult dormant metastases [21].

Here, we grouped several factors affecting the survival of patients with renal tumor thrombus into demographic, clinical, and pathological categories. Firstly, neither age nor gender was proved to have a significant influence on survival. Most studies report patients with a mean age of 56–66.5 years (range = 28–91) [18]. The mean age of patients enrolled in our paper was 64.75, while subanalysis of the cohort >75 years did not reach statistical significance either.

In our study, the following clinical factors were found to have a significant effect on patients’ prognosis: LOH, Clavien–Dindo classification, and blood transfusion. On the other hand, both Mayo stage and tumor size did not affect long-term OS. Hence, the most important controversy is that no prognostic value for the level of tumor thrombus was found, but our cohort was dominated by low Mayo stage cases. Nevertheless, when we censored the analysis at a one-year follow-up, the high Mayo stage was significantly associated with higher mortality, which is probably associated with the higher risk of perioperative complications. Other authors have emphasized the role of differences in the investigated patients between centers, constant progress in the surgical technique, the mean follow-up duration, and the particular clinicopathologic factors assessed in combination with the levels of tumor thrombus [21]. In the paper by Chen et al., no differences in cancer-specific survival (CSS) were noted when one analyzed the renal vein group and the inferior vena cava group, leading to the conclusion that the level of venous tumor thrombus is not an independent prognosis predictor [25]. Some authors have, however, found such a correlation [26], and these conflicting results may arise again from the enrollment of individuals with renal venous tumor thrombus only. A preoperative CT to assess the primary tumor and an MRI for tumor thrombus level detection are reliable and validated imaging methods in RCC [27] and were used in the analyzed cohort as well. Recent papers present the updated view that both imaging modalities possess excellent sensitivity and specificity, as far as the existence and the extent of the tumor thrombus are concerned [17]. The authors emphasize that the imaging is to be conducted within a week before surgery, especially in high-level tumor thrombus [17,18].

Moreover, OS was not affected by the ASA status. A possible explanation for this is the characteristics of the analyzed cohort, in which the vast majority of patients were ASA grade I or II. This is consistent with the majority of the available studies, with most enrolled patients being in a good general condition, e.g., ECOG 0.8 +/− 0.8 [18]. In the current cohort, open procedures were performed, with laparotomy being the most common approach. Some authors have reported laparoscopic techniques or even robotic ones to be feasible in these cases [23,28]. Furthermore, we did not observe any influence of the hospital reference on patients’ survival. It is worth emphasizing that we recruited both academic (*n* = 3) and non-academic centers (*n* = 2) with adequate surgical experience.

The following pathological features assessed in the study were proved to have an impact on patients’ survival in the univariate analysis: >pT3a tumors, and the presence of nodal and/or distant metastases. In the paper by Klatte et al., sarcomatoid features and perinephric fat invasion were the most powerful prognostic factors [29]. Chen et al. concluded that the presence of metastasis and lymph node invasion correlated significantly with a poor prognosis [25]. Furthermore, we found that high-grade cancers and the presence of tumor necrosis were additional prognostic factors. In the paper by Cho et al., tumor grade was identified as one of the most significant variables for predicting OS and CSS [30]. Some additional characteristics assessed in the literature include thrombus consistency, bland thrombus, and pathological subtype [22].

Our multivariable model predicting death included regional lymph node status, distant metastases, tumor grade, duration of hospitalization, and Clavien–Dindo grade. These findings are consistent with the results of the meta-analysis by Gu et al., in which Fuhrman grade, tumor necrosis, positive lymph node, and metastasis at surgery were significant predictors for OS [18]. This in turn may enrich the models used for the establishment of strategies in adjuvant clinical trials with systemic therapy. Moreover, according to Haferkamp et al., radical surgery may be used to prolong survival in metastatic patients when accompanied by systemic therapy, e.g., immunotherapy [31]. In our cohort, there were 36 (25%) metastatic cases, with mainly tyrosine kinase inhibitors-based adjuvant therapies implemented. Similar percentages were reported by Tang et al. in the Chinese population on 169 consecutive cases [23].

The available risk stratification models in RCC, e.g., the International Metastatic Renal-Cell Carcinoma Database Consortium (IMDC) model, are dedicated to patients who qualify for systemic therapy [6]. In a large retrospective study on metastatic RCC with venous tumor thrombus, Abel et al. identified the following adverse prognostic factors: poor-risk group, level 4 thrombi, systemic symptoms, and sarcomatoid histology [32]. These factors may facilitate the selection of M1 patients who will benefit from adjuvant therapy in the postoperative setting [1]. In the case of M0 patients, there are sparse data on the benefits of adjuvant systemic therapy. Despite high hopes, adjuvant pazopanib did not protect against the recurrence of high-risk, initially localized RCC (PROTECT study) [33]. On the other hand, pembrolizumab treatment increased disease-free survival in RCC patients of intermediate and high risk or M1, with no evidence of tumor after treatment [34]. Lastly, the neoadjuvant setting needs further clinical assessment, as in the NAXIVA study axitinib was found to downstage venous tumor thrombus in the inferior vena cava, with a direct impact on the extent of the surgical intervention [35].

The limitations of the present study mainly result from its retrospective nature and associated biases. Since this was a multicenter study with an analysis of a 7-year timeframe, there were multiple surgeons involved. The cohort was dominated by low Mayo stage cases, and some potentially important variables were unavailable for analysis, e.g., the presence of sarcomatoid features. Finally, we were unable to analyze CSS or relapse-free survival due to the lack of adequate data on these parameters.

## 5. Conclusions

In conclusion, we have identified factors influencing short- and long-term OS in RCC with tumor venous thrombus. A subgroup of patients with specific clinicopathological factors may experience durable benefits from the surgery. This includes patients without regional lymph node or distant metastases and with low tumor grades. Moreover, these factors may facilitate the appropriate selection of patients for adjuvant treatment. Importantly, short-term outcomes are better in patients with shorter hospital stays and low-grade Clavien–Dindo complications.

## Figures and Tables

**Table 1:** Basic characteristics of the study group. Abbreviations: LOH: length of hospitalization; TKI: tyrosine kinase inhibitors; MAB: monoclonal antibodies.

Characteristic		*n* (%)
Gender	Male	80 (56.3)
	Female	62 (43.7)
Age	<65 years	70 (49.3)
	>65 years	72 (50.7)
Histology	Clear cell	135 (95.1)
	Papillary	3 (2.1)
	Chromophobe	1 (0.7)
	Unspecified	3 (2.1)
Grade	1	4 (2.8)
	2	51 (35.9)
	3	48 (33.8)
	4	39 (27.5)
Tumor necrosis	Absent	83 (58.5)
	Present	59 (41.5
T	3a	120 (84.5)
	3b	12 (8.5)
	3c	3 (2.1)
	4	7 (4.9)
N	0	118 (83.1)
	1	23 (16.2)
	2	1 (0.7)
M	0	106 (74.6)
	1	36 (25.4)
R	0	115 (81.0)
	1	25 (17.6)
	2	2 (1.4)
	
Mayo stage	0	118 (83.1)
	1	8 (5.6)
	2	11 (7.7)
	3	4 (2.8)
	4	1 (0.7)
Approach	Laparotomy	84 (59.2)
	Lumbotomy	49 (34.5)
	n/a	9 (6.3)
ASA	I–II	127 (78.4)
	III–IV	31 (19.1)
	n/a	4 (2.4)
Length of hospitalization (LOH)	≤9 days	94 (66.2)
	>9 days	48 (33.8)
Clavien–Dindo classification	I	113 (79.6)
	II	16 (11.3)
	III	5 (3.5)
	IV	7 (4.9)
	V	1 (0.7)
Targeted adjuvant therapy	No	107 (75.4)
	TKI	29 (20.4)
	Interferon	1 (0.7)
	MAB	5 (3.5)

**Table 2:** Characteristics of the univariate analysis and multivariable Cox regression model. Abbreviations and explanations: high T: tumor ≥T3b; high grade: ≥G3; low Mayo stage: 0–1; LOH: length of hospitalization; short LOH: ≤9 days; low Clavien–Dindo: grade I.

	Univariate Analysis		Multivariable Model	
Feature	HR (+/− 95% CI)	*p*	HR (+/− 95% CI)	*p*
Male vs. Female	1.18 (0.69–2.03)	0.545		
Age <65 years vs. >65 years	0.70 (0.41–1.21)	0.203		
High T vs. T3a	2.38 (1.29–4.39)	0.005		
N1-2 vs. N0	4.99 (2.72–9.14)	<0.001	3.70 (1.92–7.12)	<0.001
M1 vs. M0	2.73 (1.58–4.70)	<0.001	2.13 (1.20–3.78)	0.009
Low vs. high grade	0.44 (0.24–0.81)	0.008	0.36 (0.19–0.68)	0.002
Tumor size <100 mm vs. >100 mm	0.77 (0.41–1.44)	0.421		
Tumor necrosis	2.54 (1.47–4.40)	<0.001		
R1-2 vs. R0	2.04 (1.09–3.85)	0.027		
Low Mayo stage	0.59 (0.29–1.22)	0.162		
Short LOH	0.38 (0.22–0.66)	<0.001	0.47 (0.25–0.87)	0.016
Blood transfusion	3.53 (1.93–6.44)	<0.001		
Low Clavien–Dindo	0.25 (0.14–0.45)	<0.001	0.49 (0.24–0.98)	0.047

## Data Availability

The data presented in this study are available on request from the corresponding authors.
